# Social media addiction and academic engagement as serial mediators between social anxiety and academic performance among college students

**DOI:** 10.1186/s40359-024-01635-7

**Published:** 2024-04-06

**Authors:** Qiaoxing Mou, Jie Zhuang, Qunhong Wu, Yaqin Zhong, Qianqian Dai, Xin Cao, Yuexia Gao, Qingyun Lu, Miaomiao Zhao

**Affiliations:** 1 Department of Health Management, School of Public Health, Nantong University, Nantong, Jiangsu China; 2 School of Public Health, Chengdu University of Traditional Chinese Medicine, Chengdu, China; 3 Department of Group Health Care, Affiliated Maternity and Child Health Care Hospital of Nantong University, Nantong, Jiangsu China

**Keywords:** Social anxiety, Social media addiction, Academic engagement, Academic performance, Serial mediators

## Abstract

**Background:**

Social anxiety has been shown to affect college students’ academic performance. However, the role of social media addiction and academic engagement in this association is unclear.

**Methods:**

A total 2661 college students completed a self-report questionnaire including Liebowitz Social Anxiety Scale, the Bergen Social Media Addiction Scale, the Utrecht Student Work Engagement Scale for Students, and the grade point average. Hayes’ PROCESS macro for SPSS was employed to test the serial mediation effect.

**Results:**

Results indicated that social anxiety was negatively related to academic performance, only academic engagement played a single mediating role in the relationship between social anxiety and academic performance, meanwhile social media addiction and academic engagement acted as serial mediators between social anxiety on academic performance.

**Conclusions:**

Social media addiction and academic engagement can explain the potential mechanisms of the association between social anxiety and academic performance, which have implications for devising intervention strategies to enhance the mental health and academic outcomes of college students.

## Introduction

Social anxiety, a strong emotional reaction and avoidance behavior of apprehension, nervousness, or fear of one or more interpersonal situations, is the most common psychiatric disorder other than major depression and alcohol dependence owing to its high lifetime prevalence of 12% [1]. Social anxiety usually emerges in adolescence and its duration can last for approximately 25 years [2], which encompasses important stages in the educational, social, and professional development of college students. Entering college means adjusting to a new environment in which many college students face a series of challenges, such as meeting new people, establishing positive interpersonal relationships, and completing appropriate academic tasks [3]. However, in this context, college students may not like to express their emotions and opinions publicly and may become self-conscious and doubt their abilities [4]. According to studies conducted in Europe and the United States, the incidence rate of social anxiety ranges from 10 to 40% amongst college students [5, 6]. In a study in China, 33.38% of the participants reported at least one symptom of social anxiety [7].

Individuals with social anxiety may isolate themselves and adopt avoidant behaviors to reduce feelings of anxiety [8]. Avoidant behavioral strategies, such as inability to engage in learning in class, avoidance of speaking, questioning, discussion, and other learning activities, can lead to problems including maladjustment and absenteeism that impair college students’ academic performance. Although previous studies have found that socially anxious college students exhibit low academic performance [9, 10], the underlying causes have been less explored. Thus, it is important to further explore the underlying mechanisms of how social anxiety affects academic performance to provide a reference for the management of college students’ academic performance.

Tinto’s dropout theory model proposes that the key to college completion is the degree of integration of participation in the academic and social systems within the school [11]. Moreover, college students with social anxiety may have problems with academic failure due to blocked academic and social integration [8, 12]. Through a survey of 300 college students and an analysis of grade point average (GPA) data, Parikh had found that academic engagement was positively correlated with GPA [13]. In a study of Canadian university students, social anxiety significantly and negatively predicted academic performance [14]. Russell and Topham also attributed college students’ low academic performance to the negative influence of social anxiety. Therefore, this study proposes the hypothesis.

### H1

*Social anxiety is negatively associated with college students’ academic performance.*

Social media addiction is a psychological state that involves a non-adaptive dependence on social media use that develops into behavioral addiction symptoms [15, 16]. A meta-analysis showed that the overall prevalence of social media addiction was 24% in 32 countries and regions, with college students being the focus group for addiction to social media use [17]. Social media addiction manifests itself as obsession with social media, excessive use, emotional control, and so on. This is similar to and is considered a subtype of Internet addiction [18].. Difficulties in social interaction are also necessary for addiction. According to Social Compensation Hypothesis [19] and the Compensatory Internet Use Theory [20], individuals suffering from social anxiety may choose to communicate and interact via online social media as a compensation to reduce anxiety and isolation to satisfy the desire to escape, which makes them particularly susceptible to social media addiction. Previous research had proposed that individuals who experience social anxiety are more inclined to develop addictive tendencies towards the excessive use of social media that offers more control and comfort [21, 22].Addiction to social media can lead students to spend excessive time on social media [23], with excessive access to specific websites for leisure reasons, leading to distractions from studying [24], causing a decrease in academic performance. It has also been proposed that student Facebook activity negatively predicts GPAs [25]. Hence, we propose the hypothesis.

### H2

*Social media addiction mediates the relationship between social anxiety and academic performance.*

Academic engagement is a continuous motivation to learn, characterized by a state of energy, dedication, and focus that is experienced as emotionally positive. Academic engagement is an important learning process factor that serves as an important factor in predicting academic performance [26]. Numerous studies have consistently highlighted the critical role of student engagement as a precursor to academic performance [27–29].. Notably, Salanova conducted a longitudinal study using cross-lagged correlation analyses, which revealed significant results supporting student engagement as a leading indicator of academic performance [26]. In fostering better academic engagement among adolescents, the significance of interpersonal relationships has been highlighted in previous research [30]. Conversely, social anxiety, which impairs individuals’ ability to effectively navigate social interactions [31], had been found to lead to reduced academic participation and engagement. It had previously been established that social anxiety affected classroom engagement due to limited communication with instructors [32]. Moreover, research had indicated a negative correlation between social anxiety and adolescents’ study engagement mediated through decreased intentional self-regulation and self-concept clarity [33]. Thus, social anxiety is associated with reduced academic performance through reduced academic engagement. Accordingly, we propose the hypothesis.

### H3

*Academic engagement mediates the relationship between social anxiety and academic performance.*

Academic engagement is characterized by energetic, purposeful, and sustained action [34], which may be regarded as visible and core manifestation of potential motivation [35]. One study found that Internet addiction was negatively correlated with motivation [36]. Furthermore, relevant empirical studies have shown that social media use can strongly interfere with learning [37]. Tong et al. suggested that prolonged use of social media may be detrimental to academic attention [38]. The above analysis suggests that when socially anxious individuals also have social media addiction, the interaction of these two factors will result in lower academic engagement, which in turn affects their academic performance. In other words, social media addiction and academic engagement may act as two distinct behavioral factors mediating the chain of social anxiety in relation to academic performance. In summary, this study proposes the hypothesis.

### H4

*Social media addiction and academic engagement are negatively related, with social media addiction and academic engagement serially mediating the association between social anxiety and academic performance.*

**Fig. 1:**
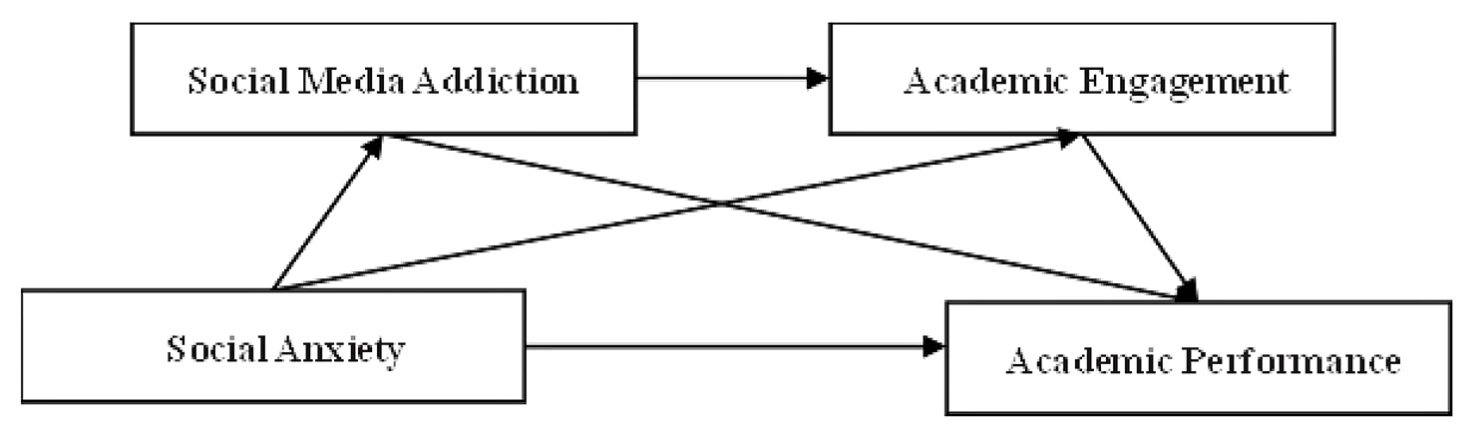
The proposed serial mediation model

In summary, this study hypothesized that social anxiety and academic engagement are predictors of college students’ academic performance, which is a significant negative correlation between social anxiety and academic performance, as well as a moderating mediator role for social media addiction and academic engagement between the two. The proposed model is shown in Fig. 1.

## Method

### Participants

The data used in this study was extracted from a larger investigation study about mental and behavior factors with health and academics, conducting from November 2020 to January 2021 at Nantong University in Jiangsu, China. Convenience sampling methods was performed. We firstly recruited 20 student investigators from all schools of main campus of the university. Then the student investigators were responsible for sending the online questionnaire by WeChat or QQ group to students of his/her school to invite them to participate on a voluntary basis. A total of 3451 students accessed the online survey and 2947 of them completed the questionnaire, with response rate of 85.4%. As the main campus of the university has about 30,000 students, our sample representative 10% of the target students. After data cleaning, 286 questionnaires with the omission of key study variables or logical errors were excluded, resulting in the inclusion of 2,661 participants in the present study (effective rate of 90.3%). Participants’ age was 19.97 ± 1.25 (ranging from 16 to 24 years). 1508 were female (56.7%), 1666 (62.6%) were non-medical students. The grades of participants are as followed: freshmen (*n* = 682; 25.6%), sophomores (*n* = 697; 26.2%), juniors (*n* = 977; 36.7%), and seniors (*n* = 305; 11.5%).

### Measurements

#### Social anxiety

Social anxiety was measured using the Liebowitz Social Anxiety Scale (LSAS) [39].The LSAS, including two subscales of fear and avoidance, is composed of 24 items including 11 items assessing social interaction and 13 items assessing performance/observation situations. Each item is rated on a 4-point Likert scale to measure the intensity of fear from 0 (*none*) to 3 (*severe fear*) and the frequency of avoidance from 0 (*never*) to 3 (*usually*). The total LSAS score is the sum of the subscale scores fear and avoidance (ranging from 0 to 144), with higher score indicating higher level of social anxiety. The Chinese version of the LSAS have been validated [40]. In the present study, Cronbach’s alpha of the LSAS was 0.973. Confirmatory factor analysis showed the four-dimensional scale had acceptable model fit (χ^2^/df = 3.26, *p* < 0.001, CFI = 0.884, TLI = 0.867, SRMR = 0.0615, RMSEA = 0.809), which were comparable with previous study [41].

#### Social media addiction

Social media addiction was measured by the Bergen Social Media Addiction Scale (BSMAS) [42],which has been widely used in previous studies [43, 44]. The BSMAS consists of 6 items, with each item rated on a 5-point Likert scale from 1 (*very rarely*) to 5(*very often*). The total BSMAS score ranges from 6 to 30, with higher scores indicating higher addiction to social media. The Chinese version of the BSMAS has been used and validated [45]. In the present study, Cronbach’s alpha of the BSMAS was 0.877.

#### Academic engagement

Academic engagement was evaluated by the Utrecht Work Engagement Scale for Students (UWES-9s) [46]. The UWES-9 S consists of 9 items assessing three dimensions of vigor, dedication, and absorption related to study. Each item of is UWES-9 S rated on a 7-point Likert scale from 0 (never) to 6 (always). The total UWES-9 S score ranges from 0 to 54, and the higher the total score, the higher the student’s academic engagement. The UWES-9 S shows good psychometric properties [47, 48]. In the present study, Cronbach’s alpha for the UWES-9 S was 0.959. Confirmatory factor analysis showed the three-dimensional scale had good model fit (χ^2^/df = 2.90, *p* < 0.001, CFI = 0.975, TLI = 0.962, SRMR = 0.025, RMSEA = 0.083).

#### Academic performance

GPA was employed to reflect academic performance. GPA can evaluate students’ learning quality in a more scientific, comprehensive, dynamic and quantitative way, which is widely used in academic performance studies [49, 50].

#### Data analyses

IBM SPSS Statistics 25.0 was used to conduct data analyses. Prior to the formal analysis, the variance inflation factor (VIF) [51] and Harman’s single-factor test [52] was used to test multi-collinearity and common method bias between the study variables. Descriptive statistics and Pearson correlation analyses were then performed. Model 6 in the SPSS PROCESS macro was used to test the serial mediation effect [53]. In regression-based analysis, effect sizes are interpreted according to Cohen’s conventions: β coefficients below 0.3 were considered small, between 0.3 and 0.5 were considered medium, and equal to or greater than 0.5 are considered large [54, 55]. Finally, bootstrap confidence interval (CI) analysis based on 5000 random resamples was performed to test the significance of the mediation effect, with the effects being considered significant if the CIs did not include zero.

## Results

### Preliminary analyses

Collinearity diagnostics showed that none of the VIF values of the study variables were > 3, and no tolerance values were < 0.3, indicating low concern for multi-collinearity issues [56]. Harman’s single-factor test showed that 11 factors had eigenvalues greater than 1, with 36.77% of the total variance explained by the largest component, which was below 50% [57]. Consequently, the common method bias was not an issue.

### Descriptive and correlational analyses

As Table 1 showed that the study variables were significantly associated with each other, providing preliminary support for our hypotheses.

**Table 1:** Means, standard deviations, and correlations for study variables

Variable	M	SD	1	2	3	4
1.Social anxiety	30.91	23.674	1			
2.Social media addiction	10.03	4.509	0.369^***^	1		
3.Academic engagement	30.30	10.499	−0.359^***^	−0.270^***^	1	
4.Academic performance	3.11	0.752	−0.187^***^	−0.133^***^	0.270^***^	1

*Note* M = mean; SD = standard deviation; ^***^p < 0.001

### Test of the serial mediation model

The results of the serial mediation model were showed in Table 2. Results revealed that social anxiety was significantly negatively associated with academic performance (*β* = -0.097, *p* < 0.001), indicating that for per one standard deviation (SD) increase in social anxiety, academic performance would decrease by 0.097 SD. Consequently, hypothesis H1 was validated. Furthermore, social anxiety showed a positive correlation with social media addiction (*β* = 0.364, *p* < 0.001), implying that social media addiction would increase 0.364 SD per one SD increase in social anxiety. However, the association between social media addiction and academic performance was not found to be significant, thus hypothesis H2 was not supported. As predicted by hypothesis H3, social anxiety was significantly negatively related to academic engagement (*β* = -0.307, *p* < 0.001). This suggested that for per one SD increase in social anxiety, academic engagement would decrease by 0.307 SD. Then, academic engagement in turn significantly positively related to academic performance (*β* = 0.219, *p* < 0.001), indicating that academic performance would increase 0.219 SD per one SD increase in academic engagement. Finally, social media addiction and academic engagement was significantly negative related (*β* = -0.160, *p* < 0.001), meaning that academic engagement would decrease 0.160 SD per one SD increase in social media addiction. Thus, hypothesis H4 was validated. Moreover, these findings indicated the presence of small and medium correlations among the study variables.

**Table 2:** The serial mediation model for relationship between social anxiety and academic performance in regression-based analysis

Criterion	Predictors	R	R²	F	β	t	95%CI
Social media addiction	Social anxiety	0.375	0.140	108.457^***^	0.364^***^	20.151	(0.329, 0.400)
Academic engagement	Social anxiety	0.401	0.160	101.506^***^	-0.307^***^	-15.996	(-0.344, -0.269)
	Social media addiction				-0.160^***^	-8.335	(-0.197, -0.122)
Academic performance	Social anxiety	0.298	0.089	43.240^***^	-0.097^***^	-4.654	(-0.138, -0.056)
	Social media addiction				-0.038	-1.878	(-0.078,0.002)
	Academic engagement				0.219^***^	10.822	(0.179,0.258)

*Note* CI = Confidence Interval. Sex, age, and academic major were as control variables. The study variables excluding control variables in regression models were standardized. ^***^p < 0.001

**Fig. 2:**
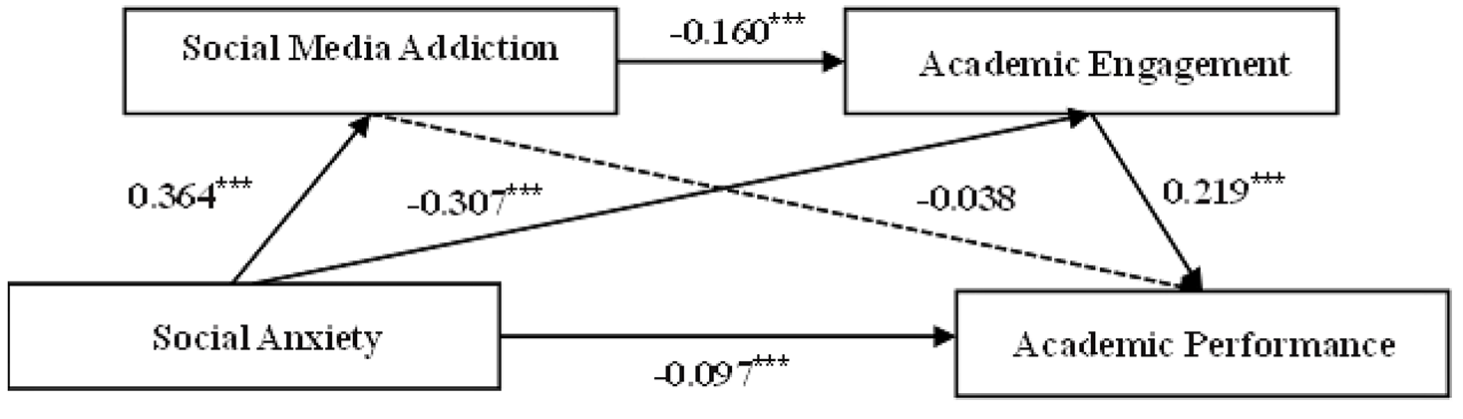
The serial mediation model of the relationship between social anxiety and academic performance. ^***^p < 0.001

The bootstrap results (Table 3) showed that all the three indirect paths were significant. The total indirect effect was − 0.094 (95% CI: -0.117 to -0.071), accounting for 49.2% of the total effect. Specifically, although the pathway of social anxiety →social media addiction → academic performance was not statistically significant (effect= -0.014, 95% CI: -0.031 to 0.003), the other specific pathway of social anxiety → academic engagement → academic performance was significant (effect= -0.067, 95% CI: -0.084 to -0.052), accounting for 35.1% of the total effect; the sequential pathway of social anxiety → social media addiction → academic engagement → academic performance was also significant (effect= -0.013, 95% CI: -0.018 to -0.008), accounting for 6.8% of the total effect. The detailed pathway model was shown in Fig. 2.

Furthermore, social anxiety has a significant direct on academic performance (effect= -0.097, 95% CI: -0.138 to -0.056), suggesting that social media addiction and academic engagement played a partial mediating role in link between social anxiety and academic performance. The serial mediation model explained 8.9% of the variance in college students’ academic performance (F = 43.240, *p* < 0.001).

**Table 3:** Social anxiety and academic performance in the mediation effect analysis

	Effect	SE	Bootstrapping 95%CI
Total effect	−0.191	0.019	(− 0.228, − 0.154)
Direct effect	−0.097	0.021	(− 0.138, − 0.056)
Total indirect effect	−0.094	0.011	(− 0.117, − 0.071)
Indirect effect (X→M1→Y)	−0.014	0.009	(− 0.031, 0.003)
Indirect effect (X→M2→Y)	−0.067	0.008	(− 0.084, − 0.052)
Indirect effect (X→M1→M2→Y)	−0.013	0.002	(− 0.018, − 0.008)

*Note* SE = Standard Error; CI = Confidence Interval. Based on 5000 bootstrap samples. Total, direct, and indirect effects of social anxiety (X) on academic performance (Y) through social media addiction (M1) and academic engagement (M2)

## Discussion

This study provided a possible explanatory mechanism for the effects of social anxiety on academic performance among college students. By examining the serial mediation pathway of social media addiction and academic engagement, the study expands on existing knowledge regarding the negative impact of mental disorders on academic life. Consistent with previous research [58–60], the findings demonstrate a significant negative correlation between social anxiety and academic performance. This aligns with self-presentation theory [61], which suggests that the characteristics of social anxiety, such as reluctance, avoidance, and fear, hinder students’ learning activities, particularly in teacher-student interactions and academic integration. These hindrances can lead students to doubt their own abilities to achieve their goals and lower their expectations of impression-related outcomes. Consequently, their task performance may be weakened, ultimately affecting their academic performance in a negative manner. Social anxiety may lead to cognitive and attentional biases that interfere with information processing, problem-solving, and decision-making [62]. Students with social anxiety may have difficulty focusing on academic tasks due to excessive worry or fear of negative evaluation, leading to decreased academic performance [63].Furthermore, social anxiety can increase stress levels and reduce self-esteem, which can have a detrimental effect on academic performance [64].

Our research has revealed that social media addiction can develop as a result of social anxiety. This finding is consistent with previous studies that have highlighted the dominant role of social anxiety in online addiction [65]. Socially anxious individuals may become excessively fixated on social media to compensate for a lack of fulfilling face-to-face social interaction. The desire for connection could be one possible explanation for this behavior [66]. Socially anxious individuals who are overly concerned with the evaluations and approvals of others may spend excessive time and energy on social media to satisfy their connection desires, ultimately leading to social media addiction [67]. Surprisingly, we did not find a significant association between social media addiction and academic performance. Previous research is not without precedents. A study among Qatari university students showed no linear relationship between social media use and academic performance [68]. This could be attributed to the fact that different individuals perceive and use social media differently [69]. Our finding suggested that while college students with social anxiety may have a higher tendency towards social media addiction, this does not necessarily translate into lower academic performance unless it is accompanied by reduced academic engagement.

This study found a significant independent mediation effect of academic engagement on the association between social anxiety and academic performance, revealing that low academic engagement due to social anxiety was associated with poor academic performance. Individuals who experience social anxiety may fear not being valued equally in interpersonal or social interactions, further inhibit their willingness to participate or engagement in the classroom [8].In contrast, the vigor of engagement, dedication and absorption are all related to the intensive effort required to learning in higher education [70]. Students who are more vigorous and dedicated to their studies are more likely to excel [71]. Therefore, students will achieve better academic performance when they have a proactive engagement toward emotional and energy.

In addition, as hypothesized, we found a significant sequential mediation effect between social anxiety and academic performance, that is, social media addiction was an antecedent of academic engagement. In a majority of college students, excessive use of social media focuses on non-academic needs. These non-targeted tasks on social media can compete with learning, which may consistently disrupt the learning process [72]. In addition, social media addiction manifests in excessive attention to social media and wasting time and energy on social platforms [73]. Combined this with the difficulties in inhibitory control associated with addiction, social media addiction leads to more attention deficit and hyperactivity disorder [74], which in turn inhibits academic engagement. Thus, when social media addiction is combined with low academic engagement, individuals with social anxiety exhibit poor academic performance.

Given the non-significant results of the single indirect effect of social anxiety on academic performance through social media addiction, our finding demonstrated that although an increase in social anxiety could lead to a higher level of social media addiction, this did not necessarily lead to decreased academic performance unless it was followed by decreased academic engagement. In other words, the negative effect of social anxiety on college students’ academic performance was more strongly related to academic engagement than social media addiction. These findings are of great importance in revealing the significant role of academic engagement that is affected by social anxiety in the academic performance of college students, which is conducive to formulating psychological and behavioral intervention strategies aimed at improving their academic achievement.

Although this study successfully established a serial mediation pathway to understand the impact of social anxiety on academic performance in college students, it is important to acknowledge that academic performance is influenced by a multitude of factors including but not limited to cognitive abilities, academic skills, pedagogical approach, study habits, motivation, and external support systems. This is a possible exaptation of small R^2^ in this study. Therefore, future research should aim to investigate the complex interplay between these factors and how they relate to academic performance in students with social anxiety to provide a more nuanced understanding of the complex factors that influence academic performance in college students with social anxiety and identify effective strategies for improving their academic outcomes.

This study had several limitations that should be considered. First, the current study was limited to students from one college in Nantong, China, and the cross-regional and cross-cultural applicability of the findings must be properly validated. Second, the present study relied only on self-reported data, and the effects of subjective bias and social desirability effects on the findings should be considered. Third, owing to the limitations of cross-sectional and non-experimental studies, no causal inferences can be made. In addiction we were unable to exclude the effects of all potential confounding variables.

## Conclusion

Our findings contribute to a better understanding of the negative effect of mental disorders on academic performance, highlighting the importance of social media addiction and academic engagement as serial mediators between social anxiety and academic performance. In addition, the negative effect of social anxiety on academic performance is more strongly related to academic engagement than to social media addiction. Findings of this study suggest that a joint psychological, behavioral and academic support intervention targeting social anxiety and social media addiction to improve academic engagement and outcomes among college students. It is recommended to incorporated personalized academic support, mentoring programs to enhance academic engagement. Additionally, interventions aimed at reducing social anxiety and social media addiction such as cognitive-behavioral techniques, exposure-based interventions, and promoting a supportive campus environment can also play a role in improving academic engagement and outcomes.

## Data Availability

The datasets used and/or analyses during the current study are available from the corresponding author on reasonable request.

## Abbreviations

GPA: grade point average
LSAS: Liebowitz Social Anxiety Scale
BSMAS: Bergen Social Media Addiction Scale
UWES-9s: Utrecht Work Engagement Scale for Students
VIF: variance inflation factor
CI: confidence interval
M: Mean
SD: standard deviation
SE: Standard Error
